# Contrast-enhanced ultrasound for differentiation of gallbladder sludge from polyp: A case report demonstrating clinical utility

**DOI:** 10.1016/j.radcr.2025.03.012

**Published:** 2025-03-26

**Authors:** Zeyad F. Elias, Stefanie Y. Lee

**Affiliations:** a Temerty Faculty of Medicine, University of Toronto, Toronto, Ontario, Canada; b Department of Medical Imaging, McMaster University; Hamilton Health Sciences, Juravinski Hospital and Cancer Centre, Hamilton, Ontario, Canada

**Keywords:** Gallbladder, Contrast-enhanced ultrasound (CEUS), Cost-effectiveness, Ultrasound, Gallbladder polyp, Gallbladder sludge

## Abstract

Gallbladder lesion characterization remains a common diagnostic dilemma in abdominal imaging, particularly when differentiating between polyps and organized sludge. Findings on conventional gray-scale ultrasound may be equivocal, especially when typical imaging features such as mobility are absent. We present a case of a 65-year-old patient who presented with a concerning nonmobile 3.7 cm gallbladder lesion on initial ultrasound assessment. Due to the superior sensitivity of contrast-enhanced ultrasound (CEUS) for blood flow compared to other imaging modalities, CEUS was able to demonstrate complete absence of enhancement within the lesion, consistent with tumefactive sludge rather than solid tissue, and averting the need for additional cross-sectional imaging. Surgical pathology following cholecystectomy confirmed these findings. This case highlights the utility of CEUS as a valuable tool in gallbladder imaging, potentially reducing healthcare costs and expediting appropriate patient care while avoiding the risks associated with other contrast-enhanced imaging modalities.

## Introduction

Gallbladder lesions represent a common diagnostic challenge in abdominal imaging, with the differentiation between neoplastic processes and benign findings having significant clinical implications [1]. While conventional gray-scale ultrasound remains the primary imaging modality for gallbladder assessment, its ability to definitively characterize lesions can be limited, particularly when typical imaging features are absent or equivocal [2,3]. Traditionally, mobility with positional changes has served as a reliable indicator for confirming that a sludge ball is not a true soft tissue lesion; however, diagnostic uncertainty arises when sludge becomes organized and demonstrates mass-like features [4].

Current imaging algorithms frequently suggest advancing to magnetic resonance imaging (MRI) for further characterization of indeterminate gallbladder lesions [5]. This approach, while effective, often results in increased imaging costs, potential delays in care due to limited resources, and barriers for patients with MRI contraindications such as cardiac pacemakers [6]. Furthermore, MRI (along with CT) carries risks associated with conventional contrast agents, particularly in patients with renal dysfunction [7].

Contrast-enhanced ultrasound has emerged as a valuable problem-solving tool in abdominal imaging, utilizing an intravenously injected microbubble contrast agent to offer real-time assessment of tissue perfusion patterns without ionizing radiation or risk of nephrotoxicity [8]. The use of ultrasound contrast agents, such as Definity (Perflutren lipid microsphere), allows for dynamic evaluation of lesion enhancement patterns, potentially obviating the need for more expensive cross-sectional imaging [1]. While CEUS has gained widespread acceptance in European and Asian practice for various applications, including gallbladder assessment, it remains underutilized in North America [9]. Recent literature suggests that CEUS may offer comparable diagnostic accuracy to MRI for gallbladder lesion characterization while providing advantages in terms of cost, availability, and patient safety [10,11].

## Case report

A 65-year-old male patient with no known risk factors for gallbladder cancer was referred for ultrasound evaluation of gallbladder lesions initially detected on outside imaging. The imaging had been performed in 2016 to investigate right upper quadrant and epigastric pain. He had a past medical history of dyslipidemia, hypertension, gastroesophageal reflux disease (GERD), obstructive sleep apnea, and osteoarthritis. He was a former smoker with a 30 pack-year history, having quit 19 years prior. His surgical history included a prior appendectomy. The patient had no relevant family history.

At the initial presentation, the patient reported intermittent right upper quadrant and epigastric pain, prompting an initial abdominal ultrasound in January 2016. This study revealed hepatomegaly with hepatic steatosis, mild gallbladder wall thickening (3.8 mm) without evidence of cholelithiasis or acute cholecystitis. Laboratory investigations showed a total bilirubin of 9 µmol/L, AST of 37 U/L, ALT of 26 U/L, ALP of 53 U/L, GGT of 136 U/L, and a random glucose level of 7.2 mmol/L. His complete cardiac workup, performed concurrently for unrelated chest pain, was unremarkable, including negative troponins, normal ECG, and a normal stress test.

**Fig. 1:**
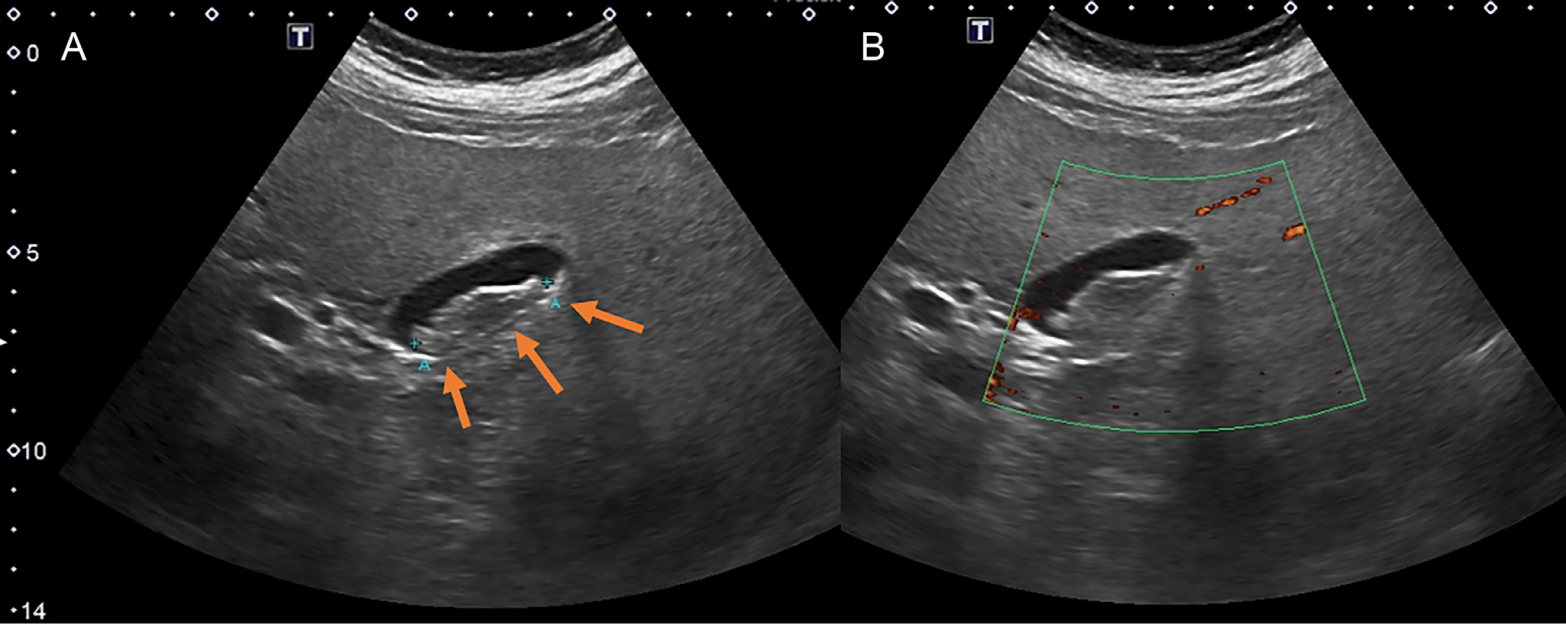
(A) Transverse grayscale ultrasound image of the gallbladder demonstrates a 3.7 cm well-circumscribed intraluminal lesion (arrows) which is isoechoic to liver parenchyma. (B) On power Doppler assessment, there is no vascularity demonstrated within the gallbladder lesion.

Conventional ultrasound performed at our hospital 2 months later revealed a 3.7 cm intraluminal gallbladder body lesion that was well-circumscribed, slightly lobulated, and isoechoic to liver parenchyma (Fig. 1). This lesion was also stable in comparison to priors. It demonstrated no mobility despite various positioning maneuvers, and revealed no vascularity on color doppler assessment. It was considered indeterminate and favored to represent a gallbladder polyp. A CEUS examination was recommended, with a triphasic CT assessment suggested as alternative.

The remainder of the ultrasound examination was unremarkable aside from focal waist-like thickening of the gallbladder wall near the fundus with small internal cystic spaces, in keeping with adenomyomatosis. The gallbladder wall was otherwise normal in thickness (<3 mm) without evidence of cholecystitis or calculi. The common bile duct measured 3 mm without evidence of biliary dilatation. The liver demonstrated mild-moderate fatty change without focal lesions.

**Fig. 2:**
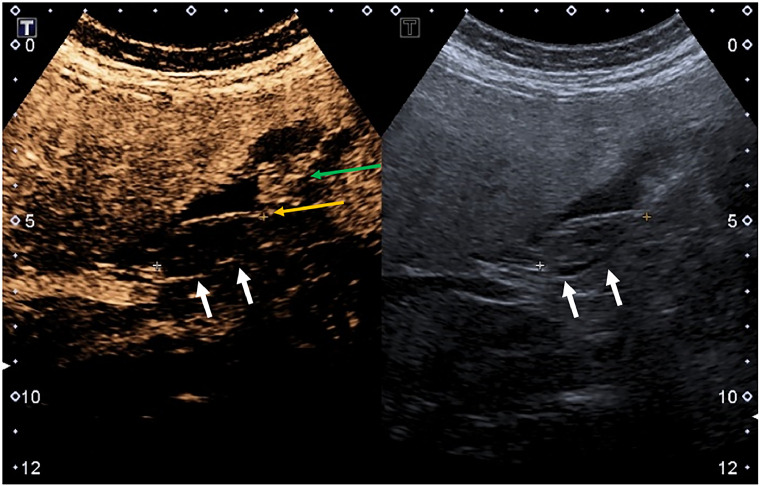
CEUS with Definity contrast demonstrates no enhancement within the gallbladder body lesion (white arrows), in keeping with tumefactive sludge. The linear echogenic line at the anterior aspect of the gallbladder lesion (yellow arrows) is present precontrast as well as on the CEUS, and is therefore in keeping with a reflective interface rather than enhancement. Incidental note is made of focal waist-like thickening near the gallbladder demonstrating enhancement with small cystic spaces on CEUS (green arrow), in keeping with adenomyomatosis.

Given the diagnostic uncertainty on the initial assessment, further characterization was warranted. CEUS was performed using Definity contrast agent (Lantheus; Billerica, MA, USA). The examination followed standardized protocols for gallbladder CEUS, including continuous imaging through arterial and venous phases [12]. CEUS demonstrated complete absence of enhancement within the gallbladder lesion throughout all phases, definitively characterizing it as a sludge ball (Fig. 2).

Given his persistent symptoms, he was advised to use a proton pump inhibitor (PPI) daily and to reduce alcohol intake to a maximum of 2 drinks per day, down from the original 5-7. Despite these measures, he continued to experience recurrent biliary colic, leading to a surgical consultation. Elective cholecystectomy was performed in late 2017. The surgical specimen consisted of a partially collapsed gallbladder measuring 6.5 cm in length with a 2.2 cm diameter and 0.2 cm wall thickness. Gross pathology revealed an aggregate of friable chalky black material measuring 3.5 × 2.0 × 1.0 cm. Microscopic examination confirmed chronic cholecystitis with organized sludge material. This was in keeping with prior episodes of cholecystitis without malignancy. Postoperatively, the patient did not report further abdominal symptoms in follow-up visits up to 2022.

## Discussion

This case illustrates several crucial aspects of modern gallbladder imaging and highlights the evolving role of CEUS in diagnostic algorithms. The primary teaching point centers on the utility of CEUS as a modality with superior sensitivity for blood flow, resulting in high specificity for nonvascular lesions such as sludge. The complete absence of enhancement on CEUS was diagnostic for sludge in this case, as true soft tissue lesions would invariably demonstrate some degree of enhancement during dynamic evaluation [13]. The correlation between imaging findings and surgical pathology in this case report strengthens the argument for broader implementation of CEUS in diagnostic algorithms for gallbladder lesions, as this may avert the need for more expensive cross-sectional imaging studies.

Recent economic analyses have demonstrated that incorporating CEUS into diagnostic algorithms for indeterminate lesions can result in substantial healthcare cost savings. A study involving 459 patients demonstrated a 406.3% cost difference between initial CEUS assessments and additional MRI examinations. CEUS was used as first-line imaging instead of CT and MRI, and correctly described 97.06% of lesions as benign or malignant, with 96.99% sensitivity and 97.09% specificity [14]. Another study, done at a single United States hospital, demonstrated a projected annual reduction in cost of up to $117,000 with the implementation of CEUS. CEUS also offers a potentially expedited definitive diagnosis, as average time to completion for outpatient examinations was found to be 5.2, 52.3, and 123.5 days for CEUS, CT, and MRI, respectively [15]. This economic benefit extends beyond direct imaging costs, as faster diagnosis can lead to more timely intervention and reduced overall healthcare utilization.

The technical advantages of CEUS over traditional contrast-enhanced imaging deserve particular emphasis. Unlike CT or MRI, which provide static images at predetermined time points, CEUS offers real-time dynamic assessment of enhancement patterns. This temporal resolution proves particularly valuable in evaluating gallbladder lesions, where the timing and pattern of enhancement can be crucial for characterization. The safety profile of ultrasound contrast agents represents another significant advantage. Unlike iodinated CT contrast or gadolinium-based MRI contrast agents, ultrasound contrast carries no risk of nephrotoxicity or nephrogenic systemic fibrosis as it is not renally excreted [7]. Furthermore, 2 large multicenter studies totaling 100,000 patients reported the incidence of anaphylactic reactions under ultrasound contrast agents to be in the range of 1:12,000 to 1:15,000. The rate of adverse events is comparable to that of common analgesics, and lower than CT and MRI contrast agents [[16], [17], [18]]. This safety profile makes CEUS particularly valuable for patients with renal dysfunction or contrast allergies, populations that traditionally present challenges for contrast-enhanced imaging.

Looking forward, this case supports the growing body of evidence suggesting that CEUS should be considered a first-line problem-solving tool for indeterminate gallbladder findings. However, it is important to note that CEUS implementation involves some considerations, including the need for specialized training, limited availability, and specific logistical considerations such as IV contrast administration—which may require ultrasound technologists to be trained in IV insertion or necessitate involvement of radiology nurses. Despite these nuanced aspects, there are resources and support available through workshops and courses hosted by societies such as the International Contrast Ultrasound Society [19]. The combination of diagnostic accuracy, cost-effectiveness, and safety makes CEUS an option to consider in the modern healthcare environment, where both clinical and economic factors must be carefully balanced.

## Patient consent

The patient discussed within this case report provided informed consent that their deidentified information may be used for educational purposes. All patient information has been anonymized to protect privacy, and no identifiable details are included.

## References

[1] Mellnick V.M., Menias C.O., Sandrasegaran K. (2015). Polypoid lesions of the gallbladder: disease spectrum with pathologic correlation [published correction appears in Radiographics. Radiographics.

[2] Zhang H.P., Bai M., Gu J.Y., He Y.Q., Qiao X.H., Du L.F. (2018). Value of contrast-enhanced ultrasound in the differential diagnosis of gallbladder lesion. World J Gastroenterol.

[3] Inoue T., Kitano M., Kudo M. (2007). Diagnosis of gallbladder diseases by contrast-enhanced phase-inversion harmonic ultrasonography. Ultrasound Med Biol.

[4] Kim M., Kang T.W., Jang K.M. (2017). Tumefactive gallbladder sludge at US: prevalence and clinical importance. Radiology.

[5] Seong M., Kang T.W., Kim M., Kim S.S., Jang K.M., Kim Y.K. (2016). Tumefactive gallbladder sludge: the MRI findings. Clinical Radiology.

[6] Pearce M.S., Salotti J.A., Little M.P. (2012). Radiation exposure from CT scans in childhood and subsequent risk of leukaemia and brain tumours: a retrospective cohort study. Lancet.

[7] Su T.H., Hsieh C.H., Chan Y.L. (2021). Intravenous CT contrast Media and acute kidney injury: a multicenter Emergency department-based study. Radiology.

[8] Girometti R., Stocca T., Serena E., Granata A., Bertolotto M. (2017). Impact of contrast-enhanced ultrasound in patients with renal function impairment. World J Radiol.

[9] Wilson S.R., Merrill C.D., Darge K., Barr R.G. (2023). Increasing CEUS utilization in the USA: a call to action for the adult and pediatric body imaging community. Abdom Radiol (NY).

[10] Negrão de Figueiredo G., Mueller-Peltzer K., Schwarze V., Zhang L., Rübenthaler J., Clevert D.A (2019). Performance of contrast-enhanced ultrasound (CEUS) compared to MRI in the diagnostic of gallbladder diseases. Clin Hemorheol Microcirc.

[11] Li S., Zhou L., Chen R. (2021). Diagnostic efficacy of contrast-enhanced ultrasound versus MRI liver imaging reporting and data system (LI-RADS) for categorising hepatic observations in patients at risk of hepatocellular carcinoma. Clin Radiol.

[12] Liu L.N., Xu H.X., Lu M.D. (2012). Contrast-enhanced ultrasound in the diagnosis of gallbladder diseases: a multi-center experience. PLoS One.

[13] Xie X.H., Xu H.X., Xie X.Y. (2010). Differential diagnosis between benign and malignant gallbladder diseases with real-time contrast-enhanced ultrasound. Eur Radiol.

[14] Smajerova M., Petrasova H., Little J. (2016). Contrast-enhanced ultrasonography in the evaluation of incidental focal liver lesions: a cost-effectiveness analysis. World J Gastroenterol.

[15] Streb J.W., Tchelepi H., Malhi H., Deurdulian C., Grant E.G. (2019). Retrospective analysis of contrast-enhanced ultrasonography effectiveness in reducing time to diagnosis and imaging-related expenditures at a single large United States County hospital. Ultrasound Q.

[16] Wei K., Mulvagh S.L., Carson L., Davidoff R., Gabriel R., Grimm R.A. (2008). The safety of definity and optison for ultrasound image enhancement: a 714 guest editorial by guest on November 16, 2015 downloaded from retrospective analysis of 78,383 administered contrast doses. J Am Soc Echocardiogr.

[17] Pisacaglia F., Bolondi L. (2006). on behalf of the Italian Society for Ultrasound in Medicine and Biology (SIUMB) Study Group on Ultrasound contrast Agents. The safety of Sonovue in abdominal applications: retrospective analysis of 23188 investigations. Ultrasound Med Biol.

[18] Darge K., Papadopoulou F., Ntoulia A. (2013). Safety of contrast enhanced ultrasound in children for non-cardiac applications: a review by the Society for Pediatric Radiology (SPR) and the International Contrast Ultrasound Society (ICUS). Pediatric Radiol.

[19] International Contrast Ultrasound Society. Archived webinars. Accessed November 19, 2024: From https://icus-society.org/education/icus-archived-webinars/.

